# Social Activities and Health-Related Quality of Life in Rural Older Adults in South Korea: A 4-Year Longitudinal Analysis

**DOI:** 10.3390/ijerph17155553

**Published:** 2020-07-31

**Authors:** JiYeon Choi, Kyeongra Yang, Sang Hui Chu, Yoosik Youm, Hyeon Chang Kim, Yeong-Ran Park, Youn-Jung Son

**Affiliations:** 1 College of Nursing, Mo-Im Kim Nursing Research Institute, Yonsei University, Seoul 03722, Korea; jychoi610@yuhs.ac (J.C.); shchu@yuhs.ac (S.H.C.); 2 School of Nursing, Rutgers, The State University of New Jersey, Newark, NJ 07107, USA; ky204@sn.rutgers.edu; 3 College of Social Sciences, Department of Sociology, Yonsei University, Seoul 03722, Korea; yoosik@yonsei.ac.kr; 4 College of Medicine, Department of Preventive Medicine, Yonsei University, 03722 Seoul, Korea; hckim@yuhs.ac; 5 Division of Silver Industry, Kangnam University, Gyeonggi-do 16979, Korea; yeongranpark@hanmail.net; 6 Red Cross College of Nursing, Chung-Ang University, Seoul 06974, Korea

**Keywords:** aging, rural health, quality of life, social participation, longitudinal studies

## Abstract

During later life, inadequate social interactions may be associated with worse quality of life in older adults. Rural older adults are prone to developing unhealthy lifestyles related to social activities, which can lead to a poorer quality of life than that enjoyed by older adults living in urban areas. This study aimed to describe longitudinal changes in social activity participation and health-related quality of life among rural older adults, exploring potential associations with changes to in-person social activity over four years. We used prospective community-based cohort data from the Korean Social Life, Health, and Aging Project (KSHAP) collected between December 2011 and January 2016. The sample included 525 older adults who completed the measure of health-related quality of life. Our results showed a significant change in health-related quality of life according to changes in participation in meeting with friends. Even though an individual’s participation in other social activities did not show significant differences in health-related quality of life, our findings imply that in-person social activities may be an important resource to encourage participation in physical activities and to develop other positive outcomes, such as a sense of belonging or satisfaction with later life, among rural older adults.

## 1. Introduction

Population aging is a rapidly growing global challenge. According to the report from the United Nations in 2019, the number of individuals worldwide, aged 65 or older will increase by almost 80% over the next three decades [1]. In Europe and North America, major industrialized parts of the world with low crude mortality and low fertility rates, more than 25% of the population is projected to be aged 65 or older by 2050 [1]. Among developed nations, South Korea is facing a particularly swift problem of population aging. By the year 2030, the percentage of older individuals (65 years or older) is projected to be near 25% in South Korea, which is higher than the proportions estimated in countries like England (21.9%), the United States (19.7%) and China (16.2%) [2]. Similar to the cases of other Western [3] and Asian countries [4], population aging is a more prominent concern in rural areas of South Korea because many younger people migrate to urban areas for education or employment, but rarely return to their home town, leaving the remaining population disproportionately older [5].

In the context of this global trend toward extended lifespans, promoting healthy aging is becoming more important than ever; that is, maintaining well-being in older age by developing and maintaining functional ability [6]. In older people, declining physical function associated with biological aging may be a natural and irreversible process [7]. Given this biological challenge, it is particularly important for older people to optimize their health-related quality of life [8,9]. Health-related quality of life is a broad, value-laden concept that reflects how individuals perceive the impact of physical and/or mental well-being on their ability to fulfill daily functioning and interactions with others [10]. A number of factors, including socioeconomic status [11,12], health behaviors [13], and living arrangements [14] are related to the promotion of healthy aging; among these, participation in social activities has been reported as an important determinant of health-related quality of life among older adults [4,15].

As they age, many older people experience changes in their participation in social activities due to major life transitions such as retirement, the death of close family or friends, or declining physical functions. Studies have reported that the reduction in social networks common among older people can increase social isolation and loneliness, which have been identified as public health concerns due to their negative impact on physical and mental health [16,17,18]. According to previous studies [19,20,21], the contribution of participating in social activities improved the physical and mental health of older people.

For understanding social activities and health status in older people, it is important to address variations in the living environment, such as access to transportation, local safety, neighborhood stability, social and local climate, and so on, due to their influence on active and healthy aging [22]. In general, living in a rural area often limits a person’s access to resources that are critical to health, such as education, jobs, or clinics and hospital facilities [23,24]. Moreover, rural areas in many countries experience more pronounced population ageing and are likely to have higher rates of poverty and greater rates of chronic diseases than urban areas [3,24]. Thus, rural health for promoting safety and a healthy life would be vital among the rural population characterized by the ageing farming population [4,22]. In South Korea, despite the contribution of economic advancement to reducing rural–urban disparities in public services and welfare programs, health inequality among rural older adults remains a major public health issue [25]. However, findings from previous studies that have examined rural–urban differences in social activities have been inconsistent [26,27,28,29]. This may result from a number of factors, such as rapidly changing rural–urban boundaries, especially in developing countries, as well as increasing diversity in social determinants of health within the older adult population. While health-related quality of life has been the main outcome of interest in research targeting the older population, few studies have longitudinally explored quality of life during the senior years, examining its association with changes to in-person social activity among community-dwelling older adult populations in rural areas [30,31,32,33]. Therefore, the main purpose of this study was to (1) describe longitudinal changes of participation in social activities and health-related quality of life and (2) to explore the associations between changes in in-person social activities and health related quality of life among rural older adults over four years.

## 2. Materials and Methods

### 2.1. Study Design and Participants

This study is a part of the Korean Social Life, Health, and Aging Project (KSHAP) project, which was a community-based, longitudinal study. The KSHAP aimed to understand current health status, trends and determinants of health, and social network characteristics among older Koreans dwelling in a rural community: Township K, Gangwha-gun, Incheon, South Korea [34]. More than 42% of residents in Gangwha-gun engage in agriculture and about 40% of the area is farmland [34]. Township K is a typical rural Korean community in which most residents live by farming [35]. In 2013, the total population of Township K was 1864 individuals from 871 families. The KSHAP study targeted the entire older adult population aged 60 years or older, as well as their spouses; the age criterion was based upon the standard of an older age pensioner set by the National Pensions Act [35]. Detailed information on the KSHAP has been provided elsewhere [34]. The survey questionnaires included questions on general sociodemographic characteristics, health history, social network characteristics, health-related quality of life, and other physical and psychosocial functions [34]. To date, the KSHAP study has completed four survey waves: Wave 1 (December 2011–March 2012, *n* = 814), Wave 2 (December 2012–March 2013, *n* = 710), Wave 3 (December 2014–March 2015, *n* = 584), and Wave 4 (December 2015–January 2016, *n* = 573). The sample for this analysis included 525 individuals who completed the measure of health-related quality of life in both Wave 1 and Wave 4.

### 2.2. Measurements

#### 2.2.1. Sociodemographic and Health Characteristics

Variables related to sociodemographic characteristics included age, gender, work status, marital status, religion, and education. Gender was dichotomized by either male or female. Age was calculated by subtracting the participant’s birth year from the year of survey. Work status was categorized as “yes” or “no.” Marital status was categorized as “living with spouse,” “separated,” “widowed,” “divorced,” or “never married.” Religion was categorized as “no religion,” “protestant,” “Catholic,” “Buddhist,” or “other.” Education was categorized as “no education,” “elementary school,” “middle school,” “high school,” or “college or higher”.

Variables related to health characteristics included Body Mass Index (BMI), smoking status, drinking habit, medical comorbid conditions, gait speed, mental status. BMI (kg/m^2^) was calculated using height and weight and then categorized according to the geriatric BMI groups with “underweight” being a BMI < 22.5 kg/m^2^, “normal” being a BMI 22.5 kg/m^2^ to 24.9 kg/m^2^, and “overweight/obese” being a BMI ≥ 25 kg/m^2^ [36]. Smoking status was categorized as “past and current smoker,” “past smoker but not now,” “have never smoked,” or “recently started smoking.” Drinking habit was categorized as “never,” “rarely,” or “once a week or more.” Regarding medical comorbid conditions, self-reported diagnosis of hypertension, hyperlipidemia, arthritis, or osteoporosis was included. Cognitive status data was obtained using the Mini-Mental Status Examination for Dementia screening (MMSE-DS) Korean version [37]. To measure gait speed, Timed Up and Go (TUG) was obtained by a trained data collector [38].

#### 2.2.2. Health-Related Quality of Life

Health-related quality of life was measured using the Short Form Health Survey, 12-item version (SF-12) [39]. Participants were asked to rate each item on a five-point Likert scale: 1 (“poor”), 2 (“somewhat poor”), 3 (“good”), 4 (“very good”), or 5 (“excellent”) [35]. The SF-12 consists of the physical component summary (PCS) and the mental component summary (MCS). The PCS and the MCS are standardized (mean  =  50, standard deviation (SD)  =  10). For each of the eight domains, the items are summed and then converted to a 0–100 scale. Higher scores indicate better physical and mental health-related quality of life.

#### 2.2.3. Social Activities

##### In-Person Social Activities

In the KSHAP study [34], in-person social activities were defined as official or unofficial participation in social activities outside of activities related to earning income. In this analysis, we included four activity types: volunteering, religious activities, hobbies, and meeting with friends. In the Wave 1 survey, participants were asked to respond either “yes” or “no” to each item. In the Wave 4 survey, participants were asked to rate the frequency in which they participated in each activity over the prior year by choosing one of the following options: 1 (“several times per week”), 2 (“once a week”), 3 (“once a month”), 4 (“several times per year”), 5 (“once or twice a year”), 6 (“fewer than once a year”), or 7 (“not at all”). For the present analysis, we recoded response options used for Wave 4 into two categories: “yes” (more than once or twice a year, collapsing the options 1 through 5), and “no” (fewer than once a year or not at all, collapsing the options 6 and 7).

##### Social Networks

Social networks were measured by network size and network density. Network size (i.e., discussion network members) refers to the number of individuals with whom participants can discuss the important topics in their lives. In the parent study, each participant was asked to list the names of a maximum of five discussion network members with whom they had interacted during the last 12 months. When the individual’s spouse was included, the maximum number of discussion networks could be six.

Network density refers to the number of actual relationships that existed among the members of an individual participant’s social network out of the total possible number of relationships [40]. For evaluation, participants were asked to indicate the frequency of their interactions with each discussion network member based on an eight-point scale that ranged from “every day” to “less than once per year.” In the parent study, if the participant reported that they “have spoken to each other at least once per week,” a relationship is assumed to exist between the two network members. Network density can range from 0 to 1. A higher score indicates better social connectedness among members within the network.

### 2.3. Ethical Considerations and Data Collection

This study was approved by the Institutional Review Board of Yonsei University (IRB Approval No.: YUIRB-2011-012-01) and conducted following the Declaration of Helsinki guidelines. All participants had the opportunity to ask questions after a full review of the study protocol with a data collector and signed a written informed consent before their participation. Trained data collectors conducted surveys via face-to-face interviews in the participants’ homes or at the local community center. Completing the survey took an average of 48 min.

### 2.4. Data Analysis

The data were analyzed using IBM SPSS Statistics for Windows, version 25 (IBM Corp., Armonk, NY, USA). Descriptive statistics were reported for all variables. Comparisons were made between Wave 1 and Wave 4 using a paired sample t-test for continuous variables and chi-square test for categorical variables. Comparisons between Wave 1 and Wave 4 were also made within each gender category using the same analysis. Individuals’ participation in each social activity from Wave 1 to Wave 4 was summarized by four change categories: (1) answered “yes” in both Wave 1 and Wave 4 (“yes + yes”), (2) answered “yes” in Wave 1 but “no” in Wave 4 (“yes + no”), (3) answered “no” in Wave 1 but “yes” in Wave 4 (”no + yes”), and (4) answered “no” in both Wave 1 and Wave 4 (“no + no”). One-way ANOVA and the Bonferroni post-hoc tests were used to compare the changes in quality of life (mental and physical) from Wave 1 to Wave 4 by the change categories in social activities. We conducted further analysis to compare gender differences in social activities with statistically significant differences in health-related quality of life. Statistical significance was set a priori as *p* < 0.05.

## 3. Results

### 3.1. Sample Characteristics

As shown in Table 1, the average age of the participants was 71.2 (Wave 1) and 75.1 (Wave 4). The majority of the participants were women (58%) and did not finish high school (87%). The mean BMI was 24.18 kg/m^2^ and about 64% of the participants were either overweight (23–24.9 kg/m^2^) or obese (>25 kg/m^2^). From Wave 1 to Wave 4, the proportion of participants who reported living with a spouse significantly decreased (76% vs. 70.35, *p* < 0.001). The proportion of participants who reported working (i.e., employed) showed a significant increase from Wave 1 to Wave 2 (73.3% vs. 75.8%, *p* < 0.001). Participants reported smoking and drinking less in Wave 4 compared with Wave 1 (*p* < 0.001). Their gait speed, measured by the Timed Up and Go test, decreased from 12.8 to 13.2 s (*p* < 0.001); however, the test result was above the normative reference of their age group (7.1–12.7 s) and below the cut-off point for high risk of falls (i.e., 14 s). Over half of participants reported hypertension (52.8% at Wave 1, 58.5% at Wave 4). The proportion of participants with major chronic conditions (i.e., hypertension, hyperlipidemia) increased from Wave 1 to Wave 4 with the exception of those with osteoporosis. Both men and women showed similar trends of change in these characteristics from Wave 1 to Wave 4.

### 3.2. Changes in in-Person Social Activities, Social Network, and Health-Related Quality of Life over Four Years

**Figure 1:**
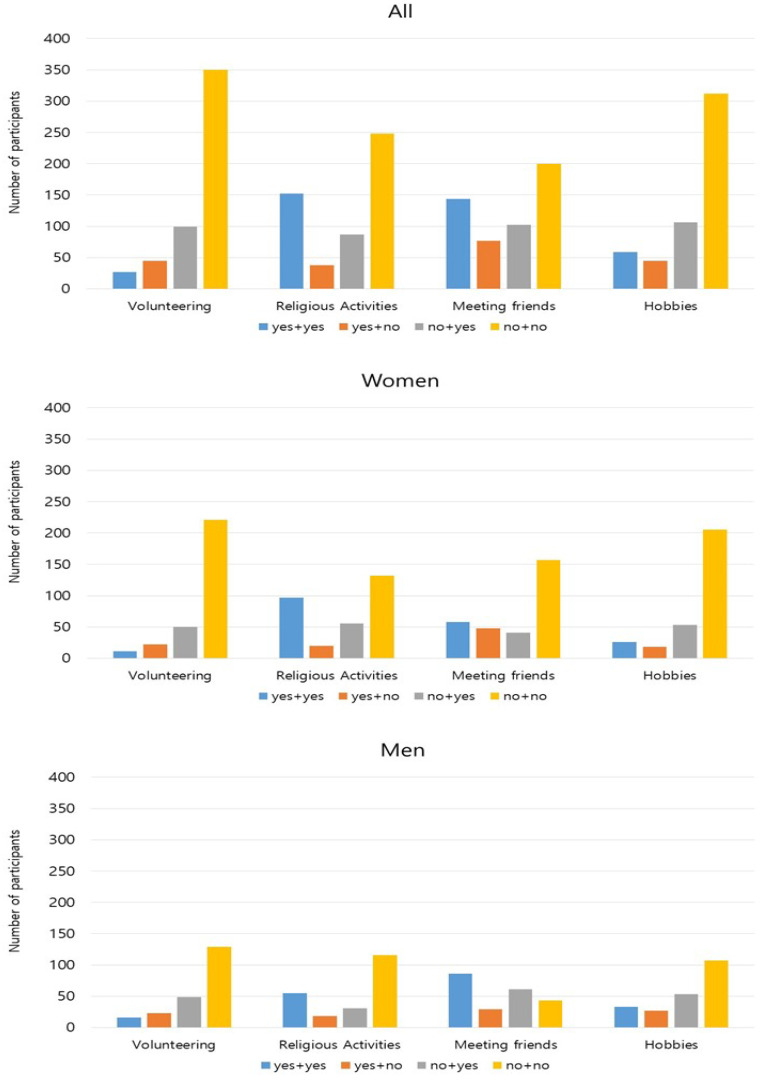
Changes in In-Person Social Activities from Wave 1 to Wave 4. Note 1: yes = participated in the listed activity; no = did not participate in the activity. Note 2: 4 change categories, yes + yes = “yes” in both Wave 1 and Wave 4; yes + no = ‘yes’ in wave 1 and ‘no’ in Wave 4; no + yes = ‘no’ in Wave 1 and “yes” in wave 4; no + no = “no” in both Wave 1 and Wave 4.

As shown in Table 2, the proportion of in-person social activity participation was higher in Wave 4 than in Wave 1 for all activities: volunteering (*p* = 0.004), religious activities (*p* = 0.001), meeting friends (*p* = 0.004), and hobbies (*p* = 0.004). These trends were consistent in both men and women with the exception of volunteering, in which more women reported participation in Wave 4 (*p* = 0.044) whereas men reported no change (*p* = 0.096). Additionally, the number of participants in each change category by social activity type is summarized in Figure 1. In all four social activities, the majority of participants showed no change in participation from Wave 1 to Wave 4 (i.e., “yes + yes” and “no + no”). This trend was consistent in both men and women (Figure 1).

Regarding social networks, there was a significant increase in discussion network size from Wave 1 (2.41) to Wave 4 (2.78; *p* < 0.001). This increasing trend was consistent in both men and women. However, there was no significant change in network density from Wave 1 (0.98) to Wave 4 (0.96; *p* = 0.178). Men and women showed different trends in the change of network density. In men, the mean network density significantly decreased from Wave 1 (0.99) to Wave 4 (0.96; *p* = 0.006) while no significant change was seen in women (*p* = 0.737).

Regarding health-related quality of life, participants’ PCS scores significantly decreased from Wave 1 to Wave 4 (*p* < 0.001). In contrast, MCS scores significantly increased from Wave 1 to Wave 4 (*p* < 0.001). These patterns of change were consistent in both men and women.

### 3.3. Changes in Health-Related Quality of Life According to Changes in in-Person Social Activities

We compared the changes in PCS and MCS scores from Wave 1 to Wave 4 among the four change categories in each social activity type. In the category of “meeting friends,” there was a significant difference in the changes in PCS and MCS scores, *F*(3, 518) = 4.275, *p* = 0.005, *F*(3, 518) = 2.813, and *p* = 0.039, respectively. Those who had previously participated but had currently stopped participating in meeting friends (i.e., the “yes + no” group) showed the highest reduction in PCS scores among the four categories (diff = −6.12) and this reduction was significantly higher than that of all other groups (*p*s < 0.05). There were no other significant differences in the changes of PCS and MCS scores among the change categories for other types of in-person social activity.

In order to identify the differences in the changes in PCS and MCS scores by the change categories in meeting friends, post-hoc comparisons were performed using the Bonferroni adjustment. Regarding the PCS, the participants who reported “yes + yes” reported a significantly greater increase than the participants in the “yes + no” category (*p* = 0.04). Participants who reported “yes + no” reported a significantly greater decrease in PCS scores than the participants in the “no + yes” category (*p* = 0.004). Moreover, participants who reported “no + no” reported a significantly greater increase in PCS score than the participants who in the “yes + no” category (*p* = 0.020). Regarding the MCS, participants who reported “yes + no” reported a significantly greater decrease than the participants in the “no + no” category (*p* = 0.037).

**Figure 2:**
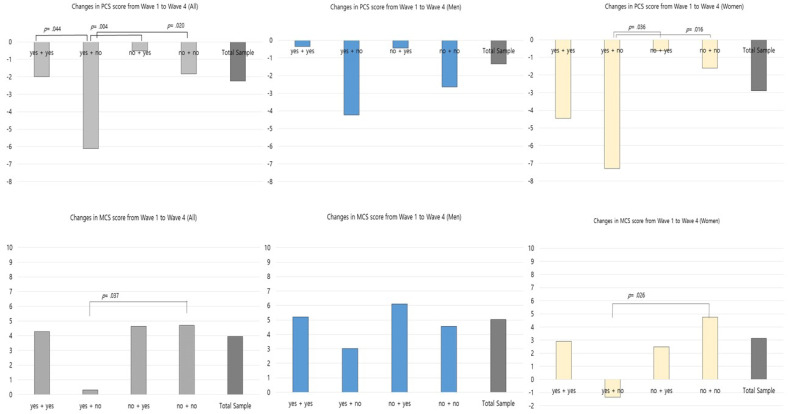
Differences in health-related quality of life between the baseline and follow-up according to changes in meeting friends. Note 1. MCS = Mental Component Summary; PCS = Physical Component Summary.

To further explore whether there was any gender difference in the effects of meeting friends, we compared the changes in PCS and MCS scores from Wave 1 to Wave 4 in men and women (Figure 2). In women, there was a significant difference in the changes in PCS and MCS scores among the change categories: *F*(3, 299) = 3.997, *p* = 0.008; *F*(3, 299) = 2.808, *p* = 0.040, respectively. The results of the post-hoc comparison revealed a significantly greater decrease in PCS scores among female participants who reported “yes + no” compared with those who reported “no + yes” (diff = −6.67, *p* = 0.036) and “no + no” (diff = −5.68, *p* = 0.016). The mean MCS score decreased in female participants who reported “yes + no,” whereas the score increased in all other categories. There was a significant difference in the MCS score in the “yes + no” category and those in the “no + no” category (*p* = 0.026) among female participants.

## 4. Discussion

An important indicator of healthy aging in older people is the preservation of good physical and mental health while living in a familiar environment [41]. In developed countries, rapid industrialization has led to economic development and improved housing, educational opportunities, and public health access in urban areas [42]. Simultaneously, however, a rapid decline in the rural population has not only affected the agricultural labor force, but also the typical family structure, such as nuclear family configurations, which may increase the risk of isolation and poorer health-related quality of life among rural older adults [42]. This study provided insights into the longitudinal change of health-related quality of life (i.e., PCS and MCS scores of SF-12) and association between changes in social activities (i.e., in-person social activities and social networks) and health-related quality of life among older adults living in a rural village in South Korea.

### 4.1. Longitudinal Changes in Health-Related Quality of Life

Regarding the longitudinal change of SF-12 scores in our sample, both female and male older adults reported a significant change over four years: participants reported a decrease in PCS scores and improvement in MCS scores. Comparing our findings on health-related quality of life with previous studies is challenging because of geographic diversity across the studies; moreover, relatively few studies have exclusively focused on rural older adults. We tried to compare our findings with studies targeting community-dwelling or rural older adults in various regions. Our findings were similar to those of one study of community-dwelling older adults in Korea by Kim et al. (2020) that reported a moderate average active aging score. The authors also reported on the three subdomain scores of active aging: scores on “safety” were the highest, followed by “health” and “participation” scores [43]. In contrast with our findings, Henchoz et al. (2019) found that the score of all domains of quality of life, including social and cultural life, health and mobility, and esteem and recognition, decreased in community-dwelling older adults [30]. Further, our results are not consistent with the findings of another previous study that reported that rural older adults might feel more loneliness and need for emotional support, as indicated by reporting worse emotional well-being compared with the self-report scores of urban participants [44]. These discrepancies may arise from several sources. For example, living arrangements may vary due to both cultural norms regarding filial responsibility and differences in community services across the selected countries and contexts, reflecting variations in the availability, cost, and quality of institutional care for older adults [43]. Future research should investigate the differences in health-related quality of life in older men and women between urban and rural areas. With respect to the components of physical and mental health, the average PCS score in our sample is slightly higher than that obtained in previous research [45,46], while the MCS is similar to the average value for six European countries (mean MCS score of 54.3) obtained using the SF-12 [46]. This higher PCS score in our sample may be due to participants’ younger age and lower prevalence of obesity compared with the samples in prior studies. Obesity in the elderly can lead to chronic diseases and can affect daily life [47]. Further studies are needed to compare the impact of obesity on health-related quality of life across regions at the global level.

Concerning gender differences, our findings supported previous studies, which reported a poorer health-related quality of life among women than among men [48,49,50]. In the present study, male older adults were more often currently employed and living with a spouse than female older adults. In addition, female older adults had a higher prevalence of chronic diseases such as hypertension, hyperlipidemia, arthritis, and osteoporosis compared with male older adults. Working status, living arrangement, and multi-morbidities may be associated with physical performance or mobility [51]. Mobility impairments might have decreased participants’ opportunities to participate in diverse social activities. To better understand these results, further investigation may be necessary to identify needs, ongoing behavior patterns, and barriers to mobility in rural older adults.

### 4.2. Changes in PCS and MCS Scores by Types of in-Person Social Activities

Importantly, we compared the changes in PCS and MCS scores of SF-12 for four years by types of in-person social activities. In our sample, “meeting friends” was the only social activity significantly associated with changes to physical and mental health-related quality of life. Ceasing to meet friends in Wave 4 was significantly associated with the largest decrease in PCS score and a small increase in MCS scores. This finding was in line with the result of a previous study which found that only informal social activities with friends were associated with increases or maintenance of life satisfaction [52]. Our result also aligns with a previous finding on the positive relationship between informal strong ties and subjective well-being [53]. In addition, this finding may support the results of previous studies which show that having multiple group memberships may lower the risk of functional disability and contribute toward maintaining mental health [54,55,56].

Interestingly, our finding revealed a greater decrease in PCS score among female participants who reported “yes + no” compared with those who reported “no + yes” or “no + no.” Furthermore, the mean MCS score decreased in female participants who reported “yes + no” while the score increased in all other categories. There is a significant difference in the MCS score in the “yes + no” category and the “no + no” category among female participants. However, in men, beginning to participate in meeting friends in Wave 4 seemed to play a positive role in improving MCS scores but this improvement was not statistically significant. Lam et al. (2018) argued that participation in multiple organizations is a psychosocial resource that protects older people from threats to their health due to changes in their social identity [57]. A decline in an individual’s social role due to advancing age—which is one of the main changes in social identity later in life—may lead to isolation. Therefore, formal or informal social activities may contribute to improving an individual’s health-related quality of life. Future studies are needed to investigate the gender differences in longitudinal changes of quality of life according to living arrangements and types of social activities.

In the present study, over four years, overall participation in social activities increased across all activity categories—volunteering, religious activities, meeting friends, and hobbies—as did the social network sizes for both men and women. This result was inconsistent with a prior study using national data in China, which compared rural and urban older adults; in that study, urban older adults had better social activity support and reported better health status than rural older adults [58]. Moreover, these findings are in line with the argument that engaging in informal social activities is resource demanding. Older adults are, on average, not only less healthy than middle-aged adults but also have fewer cognitive and motivational resources that may enable them to get involved in activities, which require major effort [59]. Recently, one study reported that strong informal ties might increase subjective well-being among rural individuals [60]. That is, rural older adults may benefit more from informal strong ties, such as visits with friends, neighbors, or relatives, than urban older adults. This may explain why rural older adults differ from urban older adults and younger adults with particular respect to engaging in informal social activities with friends [52]. With this in mind, it is important to take into account the advantages and disadvantages of living in rural or urban areas when studying the social interactions of older adults.

### 4.3. Limitations

Our findings must be interpreted cautiously because we explored associations between change in health-related quality of life scores and change in self-reported social activity rather than examining any causal relationship between social activity and quality of life change. Four years may not be long enough to observe major change in health-related quality of life and social activities because of relatively less variability in population migration, industry, and lifestyle in the rural area. Further longitudinal studies with older adults with diverse age groups will help answer this question. In addition, regarding the questions asking about in-person social activity participation, different answer options were used between Wave 1 (i.e., “yes” or “no”) and Wave 4 (i.e., 7 options, from 1 “several times per week” through 7 “not at all”). To resolve this difference, collapsing categories were necessary for Wave 4 data. Therefore, interpreting our results needs careful consideration on the potential contribution from the different answer options. In particular, it is possible that older adult participants underreported or were unaware of memory problems, which could lead to a measurement error. In the longitudinal analyses, because the selection characteristics of the participants in the follow-up interviews were clearly biased toward higher-functioning individuals, the relationships of primary interest were likely to have been attenuated. Self-reported data are subject to possible social desirability and recall bias, and solely relying on such responses might exaggerate potential relationships. Lastly, our data were from participants recruited from a single rural village in South Korea, and therefore could not represent the entire older adult population in rural South Korea or older adults in urban areas.

## 5. Conclusions

Our findings revealed that rural older adults who stop participating in social activities over the course of four years report a worsening quality of life compared with those who have never joined in social activities. Those people least likely to engage in social participation are likely to be the most vulnerable: those with low income or who are frail, the oldest among the elderly, and those in poor health face the most barriers to social participation. Thus, in rural areas, health professionals should be more vigilant in watching changes in living arrangements and health in older people, particularly considering the physical distance between health facilities and households in most rural communities.

For rural older adults who are able to participate in social activities, investing in a ride-share program including going to the grocery store and the doctor’s office would be beneficial. In addition, the social aspects of sharing meals would be beneficial for rural older adults who live alone. Mobile and wireless technologies may offer the potential of increasing connectedness for rural older adults by overcoming social participation challenges. Future research is needed to explore the lived experience of aging among rural older adults to identify practical approaches to promote physical and social activity, which may lead to improved overall health outcomes.

## Figures and Tables

**Table 1:** Characteristics of the Sample (*N* = 525).

Characteristics Categories		Wave 1			Wave 4			*p*-Value
		Total (*N* = 525) n (%)	Men (*n* = 220) n (%)	Women (*n* = 305) n (%)	Total (*N* = 525) n (%)	Men (*n* = 220) n (%)	Women (*n* = 305) n (%)	
Age (in years): Mean (SD)		71.17 (6.20)	70.90 (5.99)	71.36 (6.34)	75.1 (6.3)	74.75 (5.98)	75.30 (6.45)	N/A
BMI (kg/m^2^): Mean (SD)		24.18 (3.25)	23.97 (3.12)	24.32 (3.33)	-			N/A
BMI	Underweight (<18.5 kg/m^2^)	17 (3.6)	5 (2.6)	12 (4.3)	-			N/A
	Normal (18.5–22.9 kg/m^2^)	152 (32.2)	68 (35.6)	84 (29.9)	-			
	Overweight (23–24.9 kg/m^2^)	122 (25.8)	52 (27.2)	70 (24.9)	-			
	Obesity (>25 kg/m^2^)	181 (38.3)	66 (34.6)	115 (40.9)	-			
Education	No education	157 (29.9)	24 (11.0)	133 (44.0)	-			N/A
	Elementary school	225 (42.9)	92 (42.0)	133 (44.0)	-			
	Middle school	74 (14.1)	53 (24.2)	21 (7.0)	-			
	High school	49 (9.3)	39 (17.8)	10 (3.3)	-			
	College or higher	16 (3.0)	11 (5.0)	5 (1.7)	-			
Work Status	Yes	385 (73.3)	185 (84.1)	200 (65.6)	398 (75.8)	185 (84.1)	213 (69.8)	a < 0.001 b < 0.001 c < 0.001
	No	140 (26.7)	35 (15.9)	105 (34.4)	127 (24.2)	35 (15.9)	92 (30.2)	
Marital Status	Living with spouse	399 (76.0)	203 (92.3)	196 (64.3)	369 (70.3)	201 (91.4)	168 (55.1)	a < 0.001 b < 0.001 c < 0.001
	Separated	2 (0.4)	1 (0.5)	1 (0.3)	3 (0.6)	3 (1.4)	0 (0)	
	Widowed	121 (23.0)	13 (5.9)	108 (35.6)	147 (28.0)	15 (6.8)	132 (43.3)	
	Divorced	0 (0)	0 (0)	0 (0)	4 (0.8)	0 (0)	4 (1.3)	
	Never married	3 (0.6)	3 (1.4)	0 (0)	2 (0.3)	1 (0.5)	1 (0.3)	
Religion	No religion	295 (56.2)	135 (61.4)	160 (52.5)	244 (46.5)	120 (54.5)	124 (40.7)	a < 0.001 b < 0.001 c < 0.001
	Protestant	140 (26.7)	51 (23.2)	89 (29.2)	169 (32.2)	64 (29.1)	105 (34.4)	
	Catholic	28 (5.3)	11 (5.0)	17 (5.6)	24 (4.6)	9 (4.1)	15 (4.9)	
	Buddhism	55 (10.5)	19 (8.6)	36 (11.8)	65 (12.4)	19 (8.6)	46 (15.1)	
	Other	7 (1.3)	4 (1.8)	3 (1.0)	23 (4.3)	8 (3.6)	15 (4.9)	
Smoking Status	Past and current smoker	56 (10.7)	49 (22.3)	7 (2.3)	35 (6.7)	35 (15.9)	0 (0)	a < 0.001 b < 0.001 c = 0.947
	Past smoker but not now	91 (17.3)	91 (41.4)	0 (0)	124 (23.6)	120 (54.5)	4 (1.3)	
	Have never smoked	372 (70.9)	75 (34.1)	297 (97.4)	366 (69.7)	65 (29.5)	301 (98.7)	
	Recently started smoking	6 (1.1)	5 (2.3)	1 (0.3)	0 (0)	0 (0)	0 (0)	
Drinking Habit	Never	334 (63.6)	82 (37.2)	252 (82.6)	362 (69.0)	100 (45.5)	262 (85.9)	a < 0.001 b < 0.001 c < 0.001
	Rarely	68 (13.0)	36 (16.4)	32 (10.5)	66 (12.6)	29 (13.2)	37 (12.1)	
	Once a week or more	123 (23.4)	102(46.4)	21 (6.9)	97 (18.4)	91 (41.4)	6 (2.0)	
Hypertension	Yes	277 (52.8)	98 (44.5)	179 (58.7)	307 (58.5)	108 (49.1)	199 (65.2)	a < 0.001 b < 0.001 c < 0.001
	No	248 (47.2)	122 (55.5)	126 (41.3)	218 (41.5)	112 (50.9)	106 (34.8)	
						
Hyperlipidemia	Yes	57 (10.9)	23 (10.5)	34 (11.2)	111 (21.1)	36 (16.4)	75 (24.6)	a < 0.001 b < 0.001 c < 0.001
	No	467 (89.1)	197 (89.5)	270 (88.8)	414 (78.9)	184 (83.6)	230 (75.4)	
Arthritis	Yes	219 (41.9)	50 (22.8)	169 (55.6)	-	-	-	N/A
	No	304 (58.1)	169 (77.2)	135 (44.4)	-	-	-	
Osteoporosis	Yes	133 (25.3)	14 (6.4)	119 (39.0)	105 (20.0)	4 (1.8)	101 (33.1)	a < 0.001 b < 0.001 c < 0.001
	No	392 (74.7)	206 (93.6)	186 (61.0)	420 (80.0)	216 (98.2)	204 (66.9)	
MMSE-DS: Mean (SD)		24.25 (4.09)	25.39 (3.59)	23.43 (4.24)	24.38 (4.18)	25.40 (3.44)	23.64 (4.51)	a = 0.518 b = 0.963 c = 0.428
Timed Up and Go Test (in sec): Mean (SD)		12.56 (2.94)	12.04 (3.01)	12.94 (2.84)	13.34 (3.98)	12.70 (4.71)	13.80 (3.31)	a < 0.001 b < 0.001 c < 0.001

Note: a = comparison between Wave 1 and Wave 4 in all participants; b = comparison between Wave 1 and Wave 4 in men; c = comparison between Wave 1 and Wave 4 in women. MMSE-DS, Mini Mental Status Examination for Dementia Screening.

**Table 2:** Summary of In-Person Social Activities, Social Network, and Health-related Quality of Life from Wave 1 to Wave 4 (*N* = 525).

Variables	Categories	Wave 1			Wave 4			*p*-Value
		Total (*N* = 525) n (%)	Men (*n* = 220) n (%)	Women (*n* = 305) n (%)	Total (*N* = 525) n (%)	Men (*n* = 220) n (%)	Women (*n* = 305) n (%)	
In-Person Social Activities								
Volunteering	Yes	72 (13.8)	39 (18.0)	33 (10.9)	126 (24.0)	65 (29.5)	61 (20.0)	a = 0.004 b = 0.096 c = 0.044
	No	449 (86.2)	178 (82.0)	271 (89.1)	399 (76.0)	155 (70.5)	244 (80.0)	
Religious Activities	Yes	190 (36.2)	73 (33.2)	117 (38.4)	239 (45.5)	86 (39.1)	153 (50.2)	a < 0.001 b < 0.001 c < 0.001
	No	335 (63.8)	147 (66.8)	188 (61.6)	286 (54.5)	134 (60.9)	152 (49.8)	
Meeting friends	Yes	221 (42.3)	73 (33.2)	106 (34.9)	247 (47.0)	147 (66.8)	100 (32.8)	a < 0.001 b = 0.011 c < 0.001
	No	302 (57.7)	147 (66.8)	198 (65.1)	278 (53.0)	73 (33.2)	205 (67.2)	
Hobbies	Yes	104 (19.9)	115 (52.5)	44 (14.6)	166 (31.6)	86 (39.1)	80 (26.2)	a < 0.001 b = 0.003 c < 0.001
	No	418 (80.1)	104 (47.5)	258 (85.4)	359 (68.4)	134 (60.9)	225 (73.8)	
Social Network								
Network size (discussion network members); Mean (SD)		2.41 (1.17)	2.51 (1.18)	2.33 (1.15)	2.78 (1.38)	2.85 (1.47)	2.72 (1.32)	a < 0.001 b = 0.005 c < 0.001
Network density; Mean (SD)		0.98 (0.12)	0.99 (0.07)	0.97 (0.15)	0.96 (0.12)	0.96 (0.13)	0.97 (0.12)	a = 0.178 b = 0.006 c = 0.737
Health-related Quality of Life								
SF-12 Physical Component Summary: Mean (SD)		46.26 (8.74)	49.11 (7.92)	44.20 (8.75)	43.96 (10.90)	47.71 (9.43)	41.25 (11.10)	a < 0.001 b < 0.041 c < 0.001
SF-12 Mental Component Summary: Mean (SD)		49.09 (8.08)	49.99 (7.89)	48.44 (8.16)	52.99 (10.19)	55.01 (9.03)	51.55 (10.74)	a < 0.001 b < 0.001 c < 0.001

Note: a = comparison between Wave 1 and Wave 4 in all participants; b = comparison between Wave 1 and Wave 4 in men; c = comparison between Wave 1 and Wave 4 in women.
